# Robotic-assisted thoracoscopic surgery in children: a safe approach to remove thoracic tumors

**DOI:** 10.3389/fped.2025.1671131

**Published:** 2025-11-17

**Authors:** F. Palo, G. Brenco, M. Torre, A. Wolfler, S. Reali, S. Avanzini, G. Mattioli

**Affiliations:** 1 Pediatric Surgery Unit, IRCCS Istituto Giannina Gaslini, Genoa, Italy; 2 Department of Neuroscience, Rehabilitation, Ophthalmology, Genetics and Maternal Child Science, University of Genoa, DINOGMI, Genoa, Italy; 3 Department of Emergency, Division of Anesthesia, IRCCS Istituto Giannina Gaslini, Genoa, Italy

**Keywords:** pediatric robotic surgery, rats, pediatric thoracic tumors, minimally invasive surgery, oncological pediatric surgery

## Abstract

**Introduction:**

Robotic-assisted thoracoscopic surgery (RATS) in children remains a challenge, particularly in oncological cases. This study aims to provide practical and useful insights to enhance the safety and efficacy of this surgical approach.

**Methods:**

This is a single-center retrospective analysis conducted over a four-year period (2020–2025), including all pediatric patients (aged 0–18 years) who underwent RATS for thoracic tumor resection with a minimum follow-up of six months.

**Results:**

We reviewed 20 cases from pediatric patients who underwent RATS for the removal of thoracic tumors. One patient required a second procedure, totaling 21 surgeries. Neuroblastic tumors were the most frequently treated tumor (50%). The youngest patient was 16 months old, with a median age at surgery of 5 years (IQR: 14–4). The smallest patient weighed 11 kg at surgery with a median weight at surgery of 25 kg (IQR: 49.5–17). A maximum of four trocars were used. Selective ventilation was required only in 5 cases. The median operative time was 135 min (IQR: 100–180). The largest resected lesion measured 63 × 45 × 94 mm and was removed from a 3-year-old patient. Complete tumor resection was achieved in 19 patients. Conversion to open surgery was necessary in 4 cases (19%), primarily due to the need for manual tumor manipulation to ensure proper delineation. Two complications (10%) were recorded, both cases of chylothorax (Clavien-Dindo grades 2). Two patients died due to Ewing sarcoma recurrence, while all others are off therapy and in follow-up; five patients (25%) received adjuvant treatment after surgery.

**Discussion:**

Robotic surgery is a viable and safe option for pediatric thoracic tumors in selected cases. In our experience, the technique appeared suitable for all the types of tumors we have been treating, though broader applicability remains to be confirmed. However, RATS should be carefully considered in cases involving deeply infiltrating intrapulmonary lesions, major vascular involvement, or tumors requiring rib resection. Additionally, we believe single-lung ventilation is generally unnecessary unless intrapulmonary tumors are present.

## 1 Introduction

### 1.1 Overview of pediatric thoracic neoplasm

Thoracic neoplasms in children include tumors of the mediastinum, lungs, and chest wall (1). The majority of primary mediastinal tumors (approximately 60%–82%), are malignant (2). The most common etiology of these tumors varies depending on the patient's age and the location of the mediastinal mass. The anterior mediastinum is the most frequent site, accounting for 44% of cases, followed by the posterior (38%) and middle (20%) compartments (2). These patterns vary with age: younger children (under 2 years) are more likely to present with neurogenic tumors in the posterior mediastinum, whereas older children and adolescents more commonly present with lymphoid tumors in the anterior mediastinum (2). With regard to pulmonary tumors, the vast majority (over 90%) of lesions in children are benign (2). In fewer than 10% of cases, new pulmonary lesions may represent metastases from extrapulmonary malignancies (2). Primary lung malignancies in children are exceedingly rare accounting for less than 1% of cases (2). Most pediatric chest wall tumors are malignant, including Ewing sarcoma, neuroblastoma, metastatic osteosarcoma and rhabdomyosarcoma (2, 3). Nonetheless, several benign and infectious etiologies also occur, such as osteochondroma, hamartoma, fibrous dysplasia, and hemangioma (2, 3).

### 1.2 Advances in minimally invasive and robotic techniques

The use of minimally invasive surgery (MIS) in oncology is advancing; however, guidelines and indications for its use in pediatric patients with solid tumors remain less well-defined than in adults (4–6). In particular, the application of robot-assisted surgery in pediatric oncology is increasing (7–9), although robot-assisted thoracic surgery (RATS) continues to face specific challenges. Only a few studies have reported the use of the robotic surgical system in pediatric thoracic surgery (10), and even fewer have described their application in thoracic tumors in children (11, 12). Notably, there is limited evidence supporting the feasibility of RATS in low-weight pediatric patients, especially neonates (11). Robotic surgery offers well-established technical advantages, such as enhanced dexterity, three-dimensional vision, tremor filtration, and improved ergonomics, all contributing to greater precision, stability, and safety (10, 12). Additionally, robotic arms are designed to function within confined spaces, with minimal instrument conflict, and require less working space than traditional thoracoscopic surgery.

### 1.3 Rationale and aim of the study

Robotic technology allowed surgeons to push the boundaries of conventional thoracoscopy, but specific surgical guidelines were necessary (13). At our center, the use of robotic surgery has been progressively increased over time. Growing expertise with this technique enabled us to manage increasingly complex cases. In particular, pediatric thoracic oncology, one of the most technically challenging fields within pediatric surgery, had previously required a highly invasive open approach in most cases. The introduction of robotic-assisted surgery made it possible to perform these complex procedures using a minimally invasive approach, allowing for precise and effective dissection of thoracic tumors, even in very young patients.

The aim of this study was to report our experience with RATS for pediatric thoracic tumors and to provide practical recommendations in order to improve the safety and efficacy of this surgical approach.

## 2 Materials and methods

### 2.1 Study design and patient selection

We conducted a single-center retrospective analysis over a period of nearly five years (May 2020–March 2025). We included all RATS procedures performed for the resection of thoracic tumors in pediatric patients (age 0–18 years) with a minimum follow-up of six months. All procedures were documented, and demonstration videos were made available. RATS tumor resections were performed by three senior surgeons. The exclusion criteria were: thoracic tumor resection performed via open or thoracoscopic approach; age over 18 years at the time of surgery; and follow-up duration of less than six months. Apart from a few patients who were treated with an open surgical approach, no other exclusions were necessary, and all remaining eligible patients were included in the final analysis.

### 2.2 Preoperative assessment and indications of RATS

Preoperative assessment of tumor extent was carried out using computed tomography and/or magnetic resonance imaging. The indications for RATS were determined based on the size and location of the tumor, evaluation of surgical risk factors through imaging, and after multidisciplinary tumor board discussion.

### 2.3 Data collection and variables analyzed

Data on patient demographics, imaging at diagnosis, neoadjuvant chemotherapy, preoperative imaging findings, tumor volume, surgical technique, postoperative complications, histopathological diagnosis, adjuvant treatment, and oncological outcomes were collected.

### 2.4 Statistical analysis

Due to the small sample size, no advanced statistical methods or software were required. Descriptive data were presented as absolute numbers and percentages or medians with interquartile ranges where appropriate. For each complication identified, we documented the corresponding severity grade based on the Clavien-Dindo classification system (14), which is widely used to standardize the reporting of postoperative complications.

### 2.5 Previously published cases

Some of the cases included in the present study had been previously reported by the authors in a separate publication (15). In the current analysis, these cases were re-examined within a more specifically defined cohort to address distinct research objectives.

## 3 Results

### 3.1 Patient demographics

Between 2020 and 2025, a total of 20 thoracic tumors were resected in 20 children at our center through 21 RATS. These procedures accounted for 21% of all oncologic surgeries performed at our center and 50% of all RATS procedures in the same period. Neuroblastic tumors were the most frequent histological group (*n* = 10, 50%) (Table 1).

**Table 1:** Breakdown of our series.

Tumor's type (*n*, %)		*n* (%)
Neuroblastic tumors (10, 50%)	Neuroblastomas	5 (25%)
	Ganglioneuromas	3 (15%)
	Ganglioneuroblastomas	2 (10%)
Thymectomies (6, 30%)	Myasthenia gravis	3 (15%)
	Thymoma (operated 2 times)	1 (5%)
	Seminoma	1 (5%)
Metastatic disease (Ewing's Sarcoma and clear cell sarcoma)		3 (15%)
Neurofibroma		1 (5%)
Paravertebral lesion (negative histology)		1 (5%)
Total		20

Among all patients, 9 (45%) received neoadjuvant therapy. The median age at surgery was 5 years (IQR: 14-4). The younger patient had 16 months at surgery and the older one, 17 years. The median weight was 25 kg (IQR: 49.5-17). The smaller patient had a body weight of 11 kg, whereas the larger patient weighed 72 kg.

### 3.2 Conversion to open surgery

Four out 21 procedures (19%) required conversion to the open approach, with no emergency undocking. In two cases, conversions were necessary to manipulate the lesion for its proper delineation, including pulmonary metastasis of Ewing Sarcoma and a mediastinal ganglioneuroma strongly adherent to the vertebral bodies and posterior segment of the seventh rib. Another conversion occurred in a patient who underwent thymectomy for a mediastinal seminoma previously treated with chemotherapy which presented severe adhesions. The fourth patient had a recurrence of Ewing sarcoma in the para-aortic region, below the pulmonary hilum. The lesion was extremely friable and vascularized with minimal manipulation, making dissection and removal from adjacent structures (the aorta and pulmonary vein) extremely challenging, thus requiring conversion to open surgery.

### 3.3 Operative details

The procedure's durations are summarized in Table 2.

**Table 2:** Summary of surgical times.

Breakdown of operative times	Median time (range) minutes
Total operative time	87.5 (65–355)
Docking time	22.5 (10–45)
Console time	42.5 (30–180)

**Figure 1:**
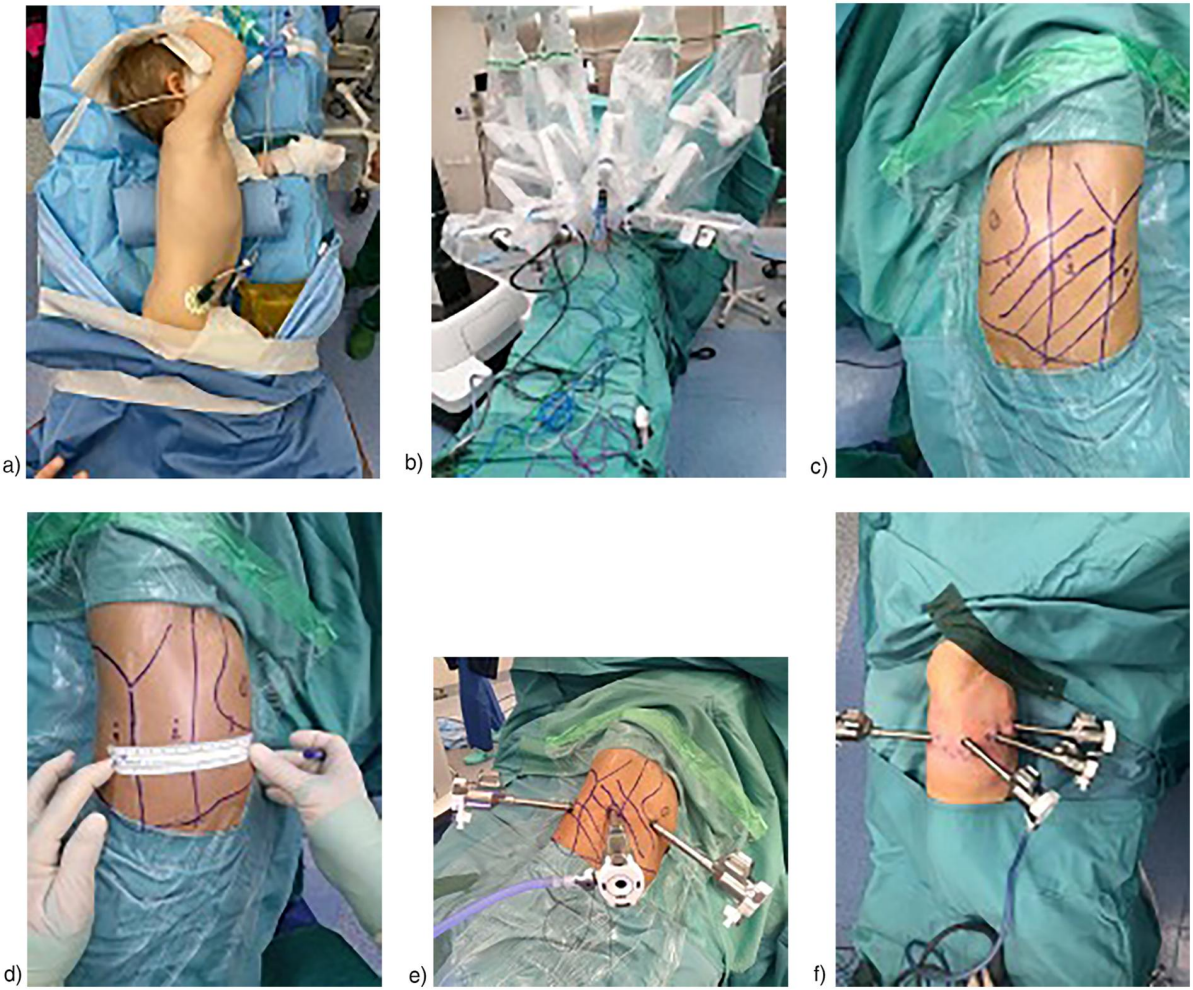
**(a)** Usual position of patients for right RATS **(b)** usual Robot's docking in RATS **(c)** 3 trocars in left RATS **(d)** 3 trocars in right RATS (distance among trocars = 6 cm) **(e)** 3 trocars in right RATS **(f)** 4 trocars in right RATS.

Among the 21 procedures, 12 (57%) were performed via a right-sided approach. The maximum number of robotic trocars used was 4, which was the setting for 14 (67%) procedures. The minimum number of robotic trocars used was 3. The trocar positions varied depending on the lesion's location. Trocar settings are summarized in Figures 1a–f. The most frequently used robotic instruments were Bipolar Maryland forceps, Cadiere forceps, monopolar scissors, Bipolar De Bakey forceps, and the Monopolar Hook. Accessory trocars were not necessary.

### 3.4 Anesthesia and ventilation management

Thoracic pressure ranged from 2 to 6 mmHg, and in most cases, this was sufficient to perform the procedure safely without requiring selective ventilation. Single lung ventilation was applied only in 5 (24%) patients, although all patients in whom it was feasible were intubated with a double-lumen tracheal tube in case it was needed. In cases where a double-lumen endotracheal tube of appropriate size was not available, such as in smaller children, a single-lumen tube was used. In these situations, a bronchial blocker was prepared and readily available; however, none of the patients required its use to complete the surgical procedure.

### 3.5 Postoperative management

A thoracic drain was placed in 19 (90%) procedures with median removal on post-operative day 3, considering only patients without complications (IQR: 4-2). The two procedures that did not require a thoracic drain were a thymectomy for myasthenia gravis and a left thoracic neuroblastoma resection in a 36-month-old patient (Table 3). Only 2 complications (10%) were recordered, both chylous effusions, which were managed conservatively with prolonged thoracic drain placement (14 days and 20 days), parenteral nutrition, and fasting, without any further surgery (Clavien-Dindo grade 2). Median hospital stay was 4 days (IQR: 3–6) in uncomplicated cases and 5 days (IQR: 3–9) when including complicated cases.

**Table 3:** Summary of the duration of post-operative chest drainage in RATS procedures for thoracic tumor excision.

Day with drain	*N* of patients
1 day	1 (5%)
2 days	6 (32%)
3 days	6 (32%)
4 days	2 (10.5%)
5 days	2 (1%)
14 days	1 (5%)
20 days	1 (5%)

### 3.6 Oncological outcomes

In 19 cases (90%), complete resection of the mass was achieved, and surgery was radical. The only exception was the patient who underwent two RATS procedures. Indeed, the patient had an unrecognized thymoma which, due to its size, could not be completely resected using the robotic approach, not even during a second procedure. A third surgery was ultimately required to achieve radical resection. The third procedure was performed via medial sternotomy. Consistent with current guidelines, the intracanalicular portion of the neuroblastic tumors was excluded from the mass isolation process and was deliberately preserved.

All patients had a minimum follow-up of 6 months. Only two deaths were recorded both due to Ewing sarcoma recurrence and disease progression. All other patients are currently in follow-up and off therapy. Five (25%) patients underwent adjuvant therapy after surgery.

All patient's data are resumed in Table 4.

**Table 4:** Summary of patients included in the study.

Patients	Diagnosis	Gender	Weigh (Kg)	Age (Months)	Thoracic distribution	Neoadjuvant Therapy	Robotic treatment	Operative time (min)	Conversion	Complications	Hospital stay (days)	Adjuvant Therapy	Outcome and Follow-Up duration
N 1	NB	M	12	36	Posterior mediastinum	Yes	NB excision	65	No	No	5	Yes	OT (3 years)
N 2	Myasthenia Gravis	F	46	135	Anterior mediastinum	No	Thymectomy	130	No	No	2	No	OT (6 months)
N 3	Myasthenia Gravis	F	48	167	Anterior mediastinum	No	Thymectomy	135	No	No	56	No	OT (3 years)
N 4	NB	M	24	68	Posterior mediastinum	Yes	NB excision	135	No	No	3	Yes	OT (4 years)
N 5	Ganglioneuroma	F	17	57	Posterior mediastinum	No	Ganglioneuroma excision	90	No	No	4	No	OT (4 years)
N 6	Lung metastasis from S. Ewing	M	22	88	Lung	Yes	Metastasis excision (atypical resections)	135	Yes	No	5	Yes	D (3 years)
N 7	NB	F	25	56	Posterior mediastinum	Yes	NB excision	75	No	No	5	No	OT (4 years)
N 8	Ganglioneuroma	M	16	36	Posterior mediastinum	Yes	Ganglioneuroma excision	170	No	No	6	No	OT (6 months)
N 9	Neurofibroma	F	47	209	Posterior mediastinum	No	Neurofibroma excision	75	No	No	3	No	OT (3 years)
N 10	Myasthenia Gravis	M	69	151	Anterior mediastinum	No	Thymectomy	130	No	No	14	No	OT (2 years)
N 11	Ganglioneuroma	F	61	64	Posterior mediastinum	No	Ganglioneuroma excision	180	Yes	No	9	No	OT (3 years)
N 12	NB	M	15	47	Posterior mediastinum	Yes	NB excision	180	No	No	4	No	OT (6 months)
N 13	Thymoma	F	51	176	Anterior mediastinum	Yes	Thymectomy	150	No	No	4	No	OT (3 years)
N 14	NB	F	11	16	Posterior mediastinum	Yes	NB excision	125	No	No	2	No	OT (2 years)
N 15	Seminoma	M	72	184	Anterior mediastinum	Yes	Thymectomy	355	Yes	Yes (C-D 2)	22	No	OT (2 years)
N 16	Lymphadenopathy	M	18	39	Posterior mediastinum	No	Paravertebral lymphadenectomy excision	80	No	Yes (C-D 2)	16	No	OT (2 years)
N 17	S.Ewing (recurrence)	M	17	56	Posterior thoracic wall	Yes	S. Ewing excision	155	Yes	No	4	Yes	D (1 year)
N 18	Ganglioneuroma	F	20.5	63	Posterior mediastinum	No	Ganglioneuroma excision	240	No	No	9	No	OT (1 year)
N 19	Ganglioneuroma	F	48	175	Posterior mediastinum	No	Ganglioneuroma excision	220	No	No	3	No	OT (6 months)
N 20	Lymphonodal metastasis from Sarcoma	M	35	138	Anterior mediastinum	No	Lymphadenectomy	110	No	No	2	Yes	OT (6 months)

N, number; M, male; F, female; NB, neuroblastoma; C-D 2, Clavien-Dindo 2; OT, off therapy; D, death due to disease progression.

## 4 Discussion

RATS for tumor resection has already been reported as safe and feasible, especially in adult surgery (6). The advantages of robotic surgery have been well established (1, 4–18). These include the enhanced range of motion of robotic instruments, elimination of the need for counterintuitive movements, tremor filtration, three-dimensional visualization, magnification of the operative field, motion scaling, and improved ergonomics for the surgeon (11). Pediatric tumors are rare and heterogeneous diseases, thus complicating the possibility to obtain evidence-based data on their minimally invasive (8). This is especially true for thoracic tumors. To date, Zeng et al. (11) have published the only large monocentric cohort of pediatric patients, reporting 149 patients with thoracic tumors excised by RATS. They reported a mean total operative time of 106 min and a conversion rate of 2.7%. The conversion rate reported in the literature ranges between 3.76% and 18.18% (11). In our study, we observed a relatively short median operative time of 135 min (IQR: 100–180), with a relatively high conversion rate (19%). However, none of the conversions were due to intraoperative complications. In both cases, these differences may be partially explained by the varying sizes of the case series, which influence surgical experience, leading to shorter operative times and reduced need for conversion. Nevertheless, our findings remain consistent with the range reported in the literature.

Indeed, at the beginning of the learning curve, it is recommended to proceed with caution and take the necessary time, as thoracic oncologic surgery is highly delicate and complex. Patient selection also plays a key role in lowering the conversion rate, and this, too, improves with experience. Nevertheless, all of our conversions were planned and performed to optimize the surgical technique, never urgently or to manage bleeding. In fact, in many cases, conversion revealed that the mass was already almost completely dissected from the surrounding structures with great precision.

One of the main concerns regarding the use of robotic systems in pediatric patients is the size of the trocars, which are larger than those used in conventional thoracoscopy (8 mm vs. 5 mm). This issue is particularly relevant in smaller children, where the limited intercostal and thoracic spaces may not easily accommodate four trocars. Nevertheless, in the majority of cases (67%), we successfully employed four trocars. In younger patients we were able to complete the procedure with only three trocars. These included four cases of mediastinal neuroblastoma, one thymic seminoma, one thymectomy in a patient with myasthenia gravis, and one pulmonary metastasectomy from Ewing's sarcoma. With the three-trocar approach, proper instrument placement was achieved without internal conflict, while minimizing chest wall stress. This suggests that minimizing the number of trocars is a viable strategy to address the limitations imposed by instrument size in small pediatric patients. By contrast, conventional thoracoscopy typically requires at least one more trocar to create a working space, which may not always be feasible in such restricted anatomical environments. Zeng et al. (11) state that thoracic robotic surgery reaches its full potential in patients older than six months and weighing more than 8 kg. In our study, which we note is based on a smaller case series, the youngest patient operated on was 16 months old and weighed 11 kg. Naturally, tumor size also plays a crucial role. According to the literature, the criteria for tumor eligibility for robotic surgery vary depending on the surgeon (11). In our series, the largest tumor measured 63 × 45 × 94 mm and was removed from a 3-year-old child. Based on our experience, we believe that there is no absolute size limit; rather, each case should be individually evaluated through imaging and multidisciplinary discussion. We are convinced that the main challenges to the robotic approach are represented by neoadjuvant therapy and the tumor's relationship with adjacent structures.

Zeng et al. (11) suggest that patients eligible for thoracoscopic tumor resection are equally suitable candidates for RATS. At our center, two different types of surgeons performed this procedure: one with prior experience in traditional thoracoscopic oncologic surgery, and one without any such background. The surgeon experienced in thoracoscopy reported that robotic resection was more precise and easier to perform than thoracoscopic surgery, due to the greater freedom of movement which represents an essential advantage in the confined space of the thoracic cavity. At the same time, for the surgeon without prior thoracoscopic oncologic experience, the robotic platform allowed for the safe and efficient execution of a complex surgical procedure.

**Figure 2:**
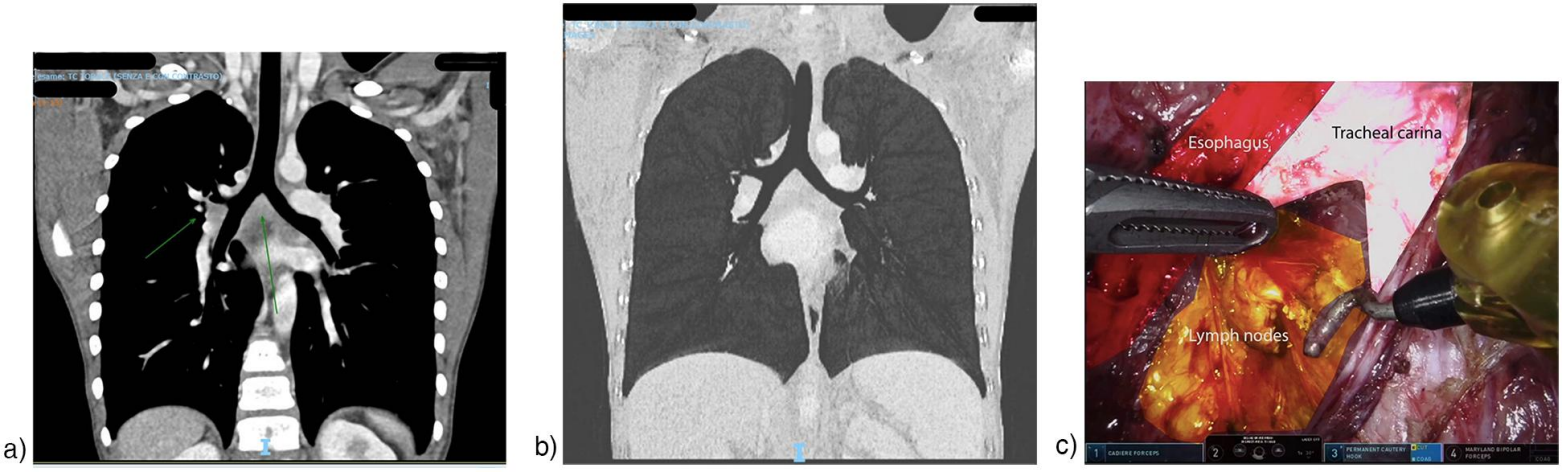
**(a,b)** preoperative computed tomography (CT) images showing the localization of the lymph nodes selected for biopsy. **(c)** Intraoperative image documenting the surgical dissection of the lymph nodes.

Furthermore, our experience confirms that the robotic approach can help streamline oncologic care (8). This is primarily due to shorter hospital stays, which help prevent delays in starting or resuming adjuvant treatments such as radiotherapy or chemotherapy. In our series, the median hospital stay was 5 days. In addition, robotic surgery enables us to perform complex procedures, such as mediastinal biopsies, using a minimally invasive technique with relative ease. For example, in our series, we treated a patient with clear cell sarcoma of the hand and lymph node metastases in the ipsilateral axilla and mediastinum, at the level of the tracheal carina Figure 2. In this high-risk, anatomically challenging area, a mediastinal biopsy was successfully performed using RATS. Without the robotic platform, the procedure would not have been feasible via conventional thoracoscopy and would have necessitated an open thoracotomy or sternotomy. The lymphadenectomy was completed efficiently, with a console time of 55 min and a total operative time of 110 min. The patient was discharged on the first postoperative day without complications, allowing prompt resumption of therapy. Without robotic assistance, minimally invasive biopsies of mediastinal masses would be technically challenging and, in some cases, would require an open approach (video available in the Supplementary Materials).

Most studies in literature emphasize the need of a selective ventilation to create adequate space within the thoracic cavity during RATS procedures (11). However, in our experience it was not necessary because the thoracic insufflation (ranged from 2 to 6 mmHg) was sufficient to establish an operative field. This is due to the precision and minimal spatial requirements of robotic instruments. Avoiding selective ventilation reduces the risk of post-surgical atelectasis which is associated with prolonged hospitalization, need of oxygen therapy and increased postoperative morbidity.

Complications during robotic-assisted thoracic surgery are uncommon but may lead to considerable morbidity and mortality if not appropriately addressed. In our case series, we observed only two instances of chylothorax. Typically, thoracic duct injury is detected postoperatively, characterized by persistently high chest tube output that becomes milky upon resumption of enteral feeding. Once chylothorax is diagnosed, established treatment protocols can be applied for management. Prompt re-exploration and thoracic duct ligation should be considered when chest tube output remains elevated (19). Although the existing literature on complications associated with robot-assisted thoracoscopic oncologic surgery is limited, our experience indicates that such complications are infrequent and generally manageable with relative ease.

## 5 Limitations

This retrospective analysis conducted at a single institution is susceptible to bias because of the absence of a control group. This study has limitations. First, the sample size is small, which may limit the generalizability of the findings and preclude robust statistical analysis. Second, the retrospective design may introduce selection and information biases. Additionally, a longer follow-up would be better to ensure the outcomes reported in the paper.

## 6 Conclusions

To the best of our knowledge, this is the largest single center retrospective study of RATS performed for thoracic tumors in Europe. Furthermore, our center is among the limited number of Italian institutions utilizing this surgical technique.

In our experience RATS is a feasible and safe surgical technique to operate oncologic thoracic diseases in children. It offers the advantages of a shorter operative time and reduced hospital stays. Nevertheless, it is necessary to take certain recommendations into account. First, the indication for robotic surgery should be limited to relatively small tumors in relation to the thoracic cavity and not involving major structures such as the heart, great vessels, or nerves. Second, patients must be carefully selected based on their neoadjuvant therapy. Lesions treated with high-dose radiotherapy and/or chemotherapy carry an increased risk of developing dense adhesions, which can make dissection more complex and riskier. These procedures should always be performed in centers with experience in thoracic and thoracoscopic surgery to ensure that the operation can be carried out safely under any circumstances. Finally, regarding weight and age limitations, in our experience, resection of thoracic neoplastic masses in children weighing less than ten kilograms is particularly complex and challenging.

Based on our experience, selective intubation is not mandatory, and the procedure is feasible also in younger children. Furthermore, RATS is a mini-invasive approach that facilitates the oncological management without delaying the timing of adjuvant therapies.

## Data Availability

The raw data supporting the conclusions of this article will be made available by the authors, without undue reservation.
